# Machine Learning Approach to Support the Detection of Parkinson’s Disease in IMU-Based Gait Analysis

**DOI:** 10.3390/s22103700

**Published:** 2022-05-12

**Authors:** Dante Trabassi, Mariano Serrao, Tiwana Varrecchia, Alberto Ranavolo, Gianluca Coppola, Roberto De Icco, Cristina Tassorelli, Stefano Filippo Castiglia

**Affiliations:** 1 Department of Medical and Surgical Sciences and Biotechnologies, “Sapienza” University of Rome, 04100 Latina, Italy; dante.trabassi@uniroma1.it (D.T.); gianluca.coppola@uniroma1.it (G.C.); stefanofilippo.castiglia@uniroma1.it (S.F.C.); 2 Movement Analysis Laboratory, Policlinico Italia, 00162 Rome, Italy; 3 Department of Occupational and Environmental Medicine, Epidemiology and Hygiene, INAIL, Monte Porzio Catone, 00078 Rome, Italy; t.varrecchia@inail.it (T.V.); a.ranavolo@inail.it (A.R.); 4 Headache Science & Neurorehabilitation Center, IRCCS Mondino Foundation, 27100 Pavia, Italy; roberto.deicco@mondino.it (R.D.I.); cristina.tassorelli@unipv.it (C.T.); 5 Department of Brain and Behavioral Sciences, University of Pavia, 27100 Pavia, Italy

**Keywords:** machine learning, artificial intelligence, gait analysis, Parkinson’s disease, harmonic ratio, K-nearest neighbors, support vector machine, random forest, artificial neural network, decision tree

## Abstract

The aim of this study was to determine which supervised machine learning (ML) algorithm can most accurately classify people with Parkinson’s disease (pwPD) from speed-matched healthy subjects (HS) based on a selected minimum set of IMU-derived gait features. Twenty-two gait features were extrapolated from the trunk acceleration patterns of 81 pwPD and 80 HS, including spatiotemporal, pelvic kinematics, and acceleration-derived gait stability indexes. After a three-level feature selection procedure, seven gait features were considered for implementing five ML algorithms: support vector machine (SVM), artificial neural network, decision trees (DT), random forest (RF), and K-nearest neighbors. Accuracy, precision, recall, and F1 score were calculated. SVM, DT, and RF showed the best classification performances, with prediction accuracy higher than 80% on the test set. The conceptual model of approaching ML that we proposed could reduce the risk of overrepresenting multicollinear gait features in the model, reducing the risk of overfitting in the test performances while fostering the explainability of the results.

## 1. Introduction

Recent advances in data analysis and wearable sensors for human movement monitoring can promote objective and accurate clinical evaluation of neurological symptoms and improve outcome measures in clinical trials [1,2,3]. Compared with optoelectronic three-dimensional motion analysis systems and instrumented walkways, wearable inertial measurement units (IMU) allow retrieval of a wide range of gait data while lowering gait analysis costs and facilitating the measurement of walking characteristics outside laboratories [1].

Gait data extracted from a single IMU in the lower back, for example, can easily define spatiotemporal gait characteristics and pelvic kinematics based on trunk acceleration patterns during walking [4]. In addition, IMU data can be used to compute trunk acceleration-derived gait indexes, which characterize the dynamic unbalance of subjects with movement disorders [5,6].

Machine learning (ML) technologies automate the study of multipurpose datasets to reveal models that would be difficult to detect using standard observation or statistical methods [7]. The automatic classification of gait impairments using ML algorithms, when combined with gait analysis using IMUs, may allow for a prompt and clinically meaningful assessment of gait abnormalities in people with movement disorders [2].

In this study, we evaluated the applicability of supervised ML algorithms for classifying gait abnormalities in people with Parkinson’s disease (pwPD) [8,9,10,11] based on IMU-derived gait parameters. pwPD suffer from gait abnormalities that closely correlate with disease progression and falls risk and affect their quality of life [12,13,14,15]. Many IMU-derived gait parameters have been shown to characterize the gait abnormalities of pwPD [5,16,17,18]. However, in studies on gait biomarkers in pwPD, a univariate approach is commonly used, in which measurement outcomes are considered independently. This approach may result in data redundancy, which is time-consuming, as well as the risk of overrepresenting intercorrelated features, which may mislead clinical interpretation [1]. The ML approach may reduce data redundancy and computational demand while also fostering clinical interpretation of these parameters by selecting the most informative gait parameters that characterize the gait of pwPD [1,16]. 

The use of ML in gait analysis has already shown promising results [19,20,21,22]. Several studies applied ML classification for the detection, quantification, and classification of gait abnormalities in pwPD using gait data from several gait analysis systems [8,9,10,11,23]. However, studies on ML on wearable sensor-derived gait data suffer a high degree of heterogeneity in model types and features introduced in the models [24], which are the two most important parameters influencing the performance of the classification algorithms. Support vector machine (SVM), decision trees (DT), random forest (RF), k-nearest neighbor (KNN), and neural networks (ANN) are the most-used supervised ML algorithms for classification purposes on wearables-derived gait data [24]. Classification models with accuracies greater than 90% have been described using these algorithms [25,26,27,28,29,30]. In general, SVM proved to be the best accurate classifier for treating gait data, whether acquired from optoelectronic or wearable sensors [31,32]. However, there is no single optimal classification tool; rather, the best algorithm performance is defined by the studied features [32,33]. In this way, RF appears to be the most robust in the case of a significant reduction in data [31], ANN is considered an adaptable algorithm with the ability to address nonlinear data [34,35], requiring a large number of parameters for a correct generalization [31], and DT and KNN benefit from dividing the problem of context recognition into smaller subproblems, which are approached one by one intuitively [32,36]. When assessing the gait of pwPD using IMUs, reducing the number of wearable devices to a single lumbar-mounted sensor allows for sufficient identification of gait abnormalities without significant information loss, reducing the wearability burden in nonlaboratory conditions [5,37,38]. Although machine learning algorithms are increasingly being used for gait analysis [39], few studies have applied ML-based classification to gait data derived from a single lumbar-mounted IMU to assess gait abnormalities of pwPD [10,36,39,40,41,42,43], and few studies have compared the classification performances of ML algorithms using lower trunk acceleration data in pwPD [36,44]. Comparing the performance of the most commonly used ML algorithms on trunk acceleration-derived gait data could provide useful information on which to use in clinical settings and which to implement into gait detection software. Rehman et al. (2019) investigated the impact of two walking protocols and two gait assessment systems on the performance of only SVM and RF, revealing that SVM performed better during continuous walking using gait data based on lower trunk accelerations. This study, however, did not provide any preliminary feature selection. Moon et al. (2020) compared the classification performances of various machine learning models in distinguishing pwPD from subjects with essential tremor, finding that ANN outperformed the other methods. However, no healthy controls were included in this study, and only clinical-based feature selection was used.

Most studies combining ML and acceleration-derived gait data attempt to use classification algorithms to reduce a large set of initial features to clinically relevant gait variables. One of the most critical challenges with supervised ML algorithms is the risk of overfitting [20,45,46]. This may happen when too many features are introduced into the algorithms in relation to the number of subjects included in the analysis. In this case, there is a high risk of obtaining a classifier learning from the specific and complete data available rather than from the underlying trend, which represents a crucial point of the ML approach. Overfitting leads to high test model performances that suffer from generalizability anyway and, thus, are not applicable in broader clinical and scientific settings [20,47,48]. Ideally, supervised ML algorithms should be trained on a set represented by a number of subjects that is approximately 20 times the number of features input into the model [47,49]. When this ratio of features to sample size is respected in studies on gait data and supervised ML, the accuracies of the retrieved models are accurate but less than 90%, reflecting the partial clinical relevance of gait parameters on the multifactorial etiology of pathologies leading to gait disorders [10,34,50]. Therefore, particularly for small samples, reducing the number of features in the model may save computational expenditure, reduce the required size of the training set to avoid overfitting [47], and simplify the medical interpretation of the classifier. Furthermore, including highly intercorrelated variables, such as gait speed and spatiotemporal gait parameters [51], may increase the risk of overrepresenting some of the variables in the test model, resulting in biased high classifier accuracies [20]. Feature selection in ML models for gait data is usually demanded by statistical approaches [35,52], using filter, wrapped, or embedded methods [53], whose combination provides the best results in classification accuracy [35,53]. In this way, because the utility of a classifier is proportional to its ability to capture the most clinically significant information, a clinical perspective should be considered during the feature selection process [47].

The main objective of this study was to identify a minimum set of speed-independent IMU-derived gait parameters to use in supervised ML algorithms and to assess the effectiveness of a feature selection procedure combining statistical methods and clinical perspectives. We also aimed to identify the supervised ML algorithm that could better fit with the preselected dataset of IMU-derived gait features in terms of accuracy, precision, recall, and generalizability. Our hypothesis was that, by reducing the number of features in the input dataset, thus lowering the risk of overfitting, and by conducting the analysis on an appropriate sample size, some of the supervised ML algorithms would show better discriminative performances than others and, thus, provide more meaningful insights into gait abnormalities in the context of a multifactorial condition such as PD.

## 2. Materials and Methods

### 2.1. Subjects

We collected data samples from 81 subjects diagnosed with idiopathic PD (25 females and 56 males, aged 72.1 ± 6.7 years) who were enrolled at “ICOT” in Latina, Italy. The following inclusion criteria were used: (i) diagnosis of idiopathic PD according to UK bank criteria [54], (ii) Hoehn and Yahr (HY) stages 1–3 [55], (iii) ability to walk independently for at least 30 m along a laboratory pathway without exhibiting freezing of gait, and (iv) a stable and accustomed drug program for at least 2 weeks before baseline. The following were the exclusion criteria: (i) cognitive deficits (defined as a score of less than 26 on the Mini-Mental State Examination) [56,57], (ii) moderate or severe depression (defined as a score of greater than 17 on the Beck Depression Inventory) [58,59], and (iii) orthopedic and/or other gait-influencing diseases, such as other neurological diseases, clinically defined osteoarthritis [60,61,62], or total hip joint replacement. Subjects who reported pain in the hip or knee joints, as well as limited range of motion in internal hip rotation, hip flexion, or visible anatomic alterations of the joints, were not included in the final analysis [62]. A group of 80 HS (aged 58.0 ± 11.0 years) was included for comparison. A 1:1 optimal matching procedure using the propensity score difference method was conducted to match pwPD and HS [63]. Each HS walked twice at self-selected and slower directed speeds to reduce the influence of gait speed on the other speed-dependent gait parameters and to collect the largest sample size possible for speed-matched comparisons [64,65]. Logistic regression analysis with age and gait speed as covariates [66,67,68] was used to obtain the propensity scores for each HY subgroup. The effectiveness of the matching procedure was confirmed using an independent sample *t*-test. The final sample included 64 pwPD (22 females, 42 males; aged 71.7 ± 6.1 years; mean disease duration 7.7 ± 4.9 years; 18 HY 1, 25 HY 2, 27 HY 3; mean UPDRS-III score 37.9 ± 14.8) and 64 speed-matched HS (HS_matched_) (33 females, 31 males, aged 58.6 ± 9.9 years).

All participants gave their informed consent in accordance with the Helsinki Declaration, and the study was approved by the local ethics committee (CE Lazio2 protocol no. 0053667/2021).

### 2.2. Instrumentation

The data were collected using an inertial sensor (BTS GWALK, BTS, Milan, Italy) fastened to an ergonomic belt around the pelvis at the level of the L5 vertebra and connected to a laptop through Bluetooth wireless standard. The sensor includes a triaxial accelerometer (16 bit/axes), triaxial magnetometer (13 bits), and triaxial gyroscope (16 bit/axes). Linear trunk accelerations and trunk angular velocities in the anterior-posterior (AP), mediolateral (ML), and vertical (V) directions were measured at a sampling rate of 100 Hz [69].

### 2.3. Task Description

Before the experiment, the participants were asked to walk on the ground along the pathway to acquaint themselves with the method. Both the pwPD and the HS were asked to walk through a corridor at their own pace. Because this study focused on natural locomotion, we only provided general and qualitative instructions, allowing participants to choose their own speed without imposing externally provided sensory cues.

### 2.4. Inertial Sensor Data Processing

The “Walk+” protocol of the G-STUDIO software (G-STUDIO, BTS, Milan, Italy) was used to detect trunk acceleration patterns, right and left heel strikes, toe-off, spatiotemporal parameters, and pelvis kinematics. Briefly, the following gait parameters were calculated: stance phase, swing phase, single support, double support, cadence, stride time, stride length, %stride length, gait speed, pelvic tilt, pelvic obliquity, pelvic rotation, coefficient of variation of step length (CV), harmonic ratio (HR), %recurrence from recurrence quantification analysis (RQArec), %determinism from recurrence quantification analysis (RQAdet). HR, RQArec, and RQAdet were calculated in the anteroposterior (ap), mediolateral (ml), and vertical (v) directions. The trunk acceleration-derived gait indexes were calculated using MATLAB (MATLAB R2021b, MathWorks, Natick, MA, USA) [5]. Python (Python 3.7.11, Python Software Foundation) was used to manage the machine learning algorithms, and statistical analyses were performed using the IBM SPSS ver. 27 and NCSS 2019 software.

### 2.5. Features Selection

Firstly, we used an independent sample *t*-test to identify the gait parameters that significantly distinguished pwPD from HS_matched_ from a dataset of 22 IMU-derived gait parameters. Assumptions were verified through the Shapiro–Wilk test. Afterward, to avoid multicollinearity, we calculated partial Pearson correlation coefficients between the gait parameters that differed between pwPD and HSmatched, removing the effects of age and gait speed. We selected the variables that showed fair-to-low (r < 0.5) correlations with the others. Among the variables that showed r ≥ 0.5, we kept those considered more clinically meaningful, based on domain expertise in movement disorders provided by a neurologist and a physical therapist and evidence from the literature [5,10,16].

Moreover, we implemented a sequential backward selection (SBS) approach to assess the importance of the selected gait variables. SBS leads to a reduction in the dimensionality of the initial feature subset while preserving classifier performance in order to enhance overall computing efficiency [70]. To implement SBS, a criterion function J to reduce for identifying the features to delete at each stage must be established. The difference between the performance of the classifier before and after the removal of each feature was used, as the criterion derived from the function J.

SBS is calculated through a four-step procedure:

1. Set the initial value for the algorithm as the chosen number of features (k) = d, where d is the dimensionality of the complete feature space *X_d_*.
2. Find the $x^{-}$ characteristic that maximizes the criterion $x^{-}$ $=argmaxJ\;\left(X_{k}-x\right)$.
3. Remove the $x^{-}$ characteristic from the set: $X_{k}-1=X_{k}-x^{-},\;k=k-1$.
4. The procedure ends if *k* equals the desired number of characteristics. Otherwise, return to step 2. The pseudocode for SBS is described in the Supplementary Materials.

We also used the random forest approach to select the features based on their importance, as their ability to decrease estimated impurity across all decision trees in the dataset.

**Figure 1:**
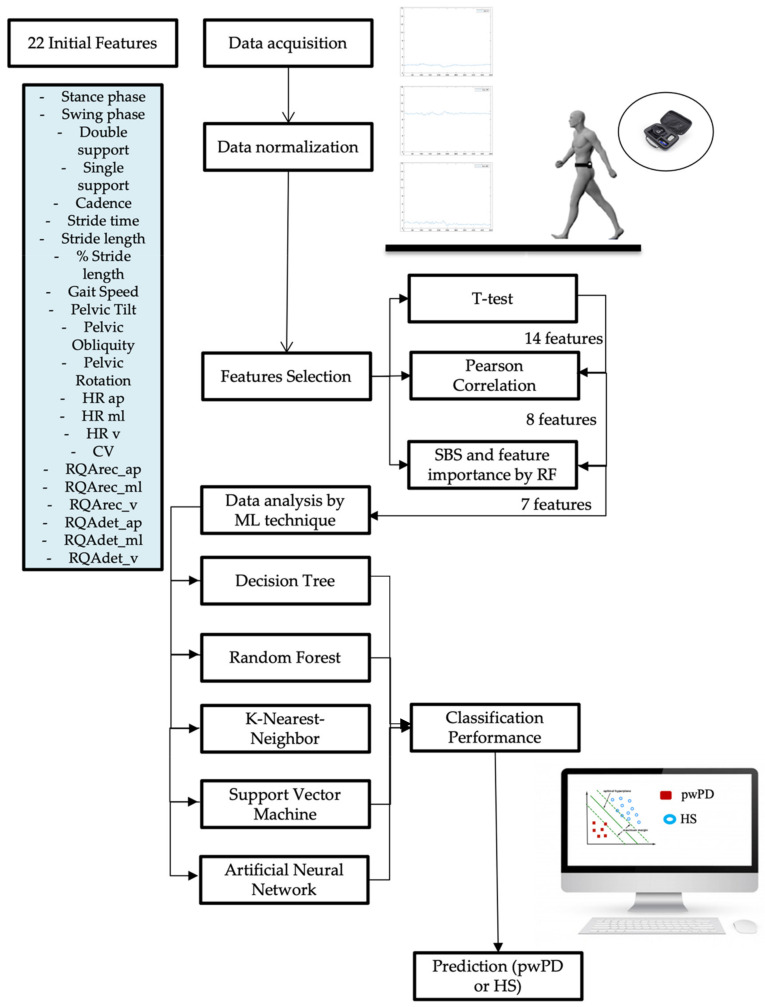
Analysis of our gait prediction flowchart. Description of the Parkinsonian gait prediction flow chart analyzed, including data acquisition, dimensionality reduction, data analysis technique, and gait prediction with classification performance parameter in prediction. First of all, data acquisition by IMU sensor positioned at the trunk level (L5) with the acceleration signals of the trunk in three spatial directions. After normalizing the data, we reduced the number of features from the initial 22 to 7, using the three methods described in feature selection. After this, we implemented five ML algorithms and evaluated their performance in prediction subjects with Parkinson’s from healthy subjects: IMU, inertial measurement unit; ML, machine learning; pwPD, people with Parkinson’s disease; HS, healthy subjects.

After features selection, we implemented the five supervised ML algorithms to assess their ability to classify the gait of pwPD from HS_matched_, evaluating a specifical flowchart for our model (Figure 1).

### 2.6. Machine Learning Model

After the data were rescaled using z score normalization [71], we used the “mean imputation” interpolation technique to replace each NaN value with the relative average obtained independently for each column of attributes in our machine learning model. We encoded the class labels as an array of integers to avoid technical misconceptions. After reducing the number of features included in the dataset, to test the learning performance of the models, we split it into two separate subsets: a training set (80% of the initial dataset) and a test set [45] (20% of the initial dataset). We considered utilizing a cross-validation technique [72] to gain reliable estimates of the model’s generalization error in order to identify a balanced trade-off between bias and variance. We implemented k-fold cross-validation, which divides the training dataset into k parts without re-entry and uses k-1 parts for model training and one part for testing. To obtain the k models and performance estimates, this technique is repeated k times [73]. The averaged performance of the models is then computed based on the many independent subdivisions, yielding a performance estimate that is less sensitive to training data partitioning than other cross-validation techniques, such as the holdout model [72]. K-fold cross-validation is also typically used to identify the ideal set of hyperparameters that provide acceptable generalization performance [74]. We used a grid search as a hyperparameter optimization method in our model, which simply performs a full search on a subset of the training algorithm’s hyperparameter space. Grid search has many dimensional gaps, but it is usually easy to parallelize because the values of the hyperparameters the algorithm works with are usually independent of each other [75].

**Figure 2:**
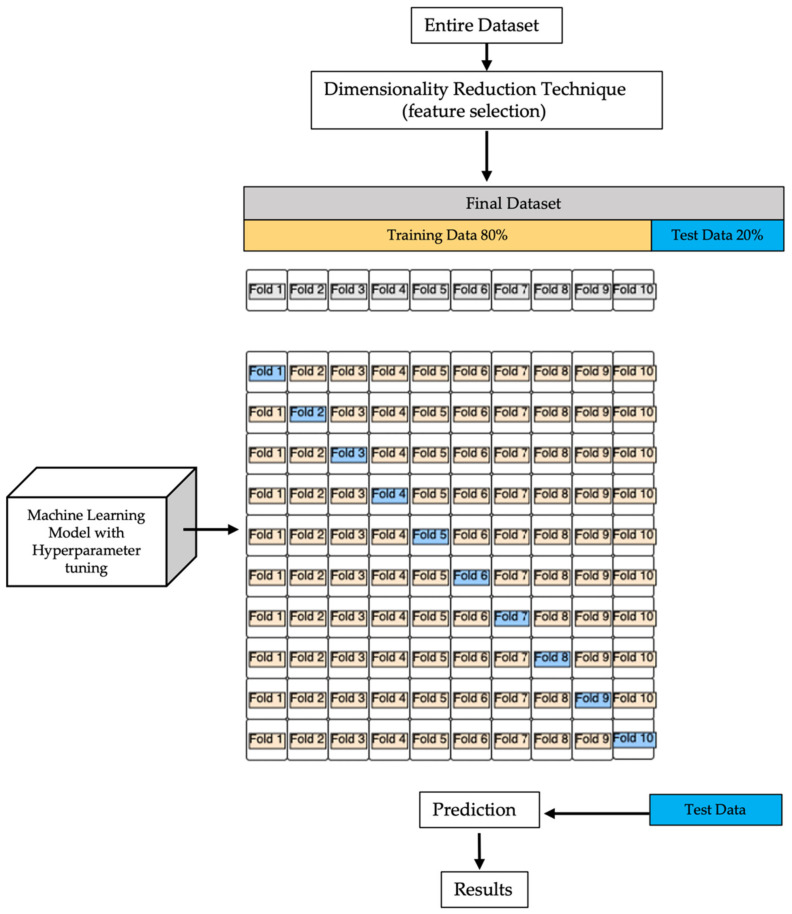
Machine learning block diagram model. After we reduced the dimensionality of the entire initial dataset, we split the final set into two subgroups: training set (80% of data) and test set (20% of data). On training data, we performed k-fold cross-validation (k = 10), as shown in the diagram. To make a prediction on training data, after grid search hyperparameter tuning, we evaluated the real output (test data), and we analyzed the results by classification performance for each classifier.

We manually specified a range of possible parameters, and the algorithm conducted a thorough search. Once the hyperparameters were verified to be satisfactory, we retrained the model on the complete set and obtained a final estimate using the independent test set. Because k-fold cross-validation is a resampling strategy without re-insertion, each sample point will only appear once in the training or the test datasets, as shown in Figure 2, resulting in a reduced variance estimate of the model’s performance.

#### 2.6.1. Tree-Based Algorithms

Decision trees (DT) are one of the most extensively used strategies for categorization problems. Because of their interpretability, DT are commonly used in machine learning. The conditional flow structure of the DT makes it simple to understand. DT partitions the feature space recursively and labels each partition based on the training data. The tree is then used to classify future points based on the splits and labels. The main advantage of DT over other methods is that they are more interpretable, which is generally preferred in many applications, such as healthcare, over other methods that may be more accurate but are more difficult to understand [76,77].

A decision tree is a classification algorithm that divides the instance space in a recursive manner. The nodes in the decision tree form a rooted tree, which is a directed tree with no incoming edges and a “root” node. In the case of numeric properties, the condition refers to a range. A class is assigned to each leaf based on the best target value. Alternatively, the leaf could contain a probability vector indicating the likelihood of the target characteristic having a particular value. Based on the results of the tests along the path, instances are classified by traveling them from the root of the tree down to a leaf [78].

In the case of numeric characteristics, DT can be considered as a collection of hyperplanes, each orthogonal to one of the axes. The decision points of the tree were chosen recursively from top to bottom in our case. This approach, also known as “divide and conquer” [79], is similar to a traditional flowchart in which if Yes, then A, and if No, then B is followed.

In the majority of cases, discrete splitting functions are univariate. A split in an internal node based on the value of a single attribute is referred to as “univariate”. As a result, the inducer seeks out the best attribute on which to split the data. There are numerous univariate criteria from which to choose. According to the origin of the measure: information theory, dependence, and distance. According to the structure of the measure: impurity-based criteria, normalized impurity-based criteria, and binary criteria. We used grid search to optimize two hyperparameters: the impurity-based criterion and the maximum depth of the tree. The best accuracy was obtained by building our decision tree with a maximum depth of five and the entropy impurity criterion.

To divide a node, decision tree algorithms leverage information gain. A node with more than one class is impure, whereas a node with only one class is pure. If a node contains several classes, it is considered disordered. Entropy was calculated as follows [80]:

$$Entropy=\overset{n}{\underset{i=1}{\sum }}-p(c_{i})log_{2}p\left(c_{i}\right)$$

where $p(c_{i})$ is the probability of class $c_{i}$ in a node, and n is the number of data points.

Another widely used tree-based classification strategy is represented by random forests (RF). RF is a classification method that employs a large number of decision trees and can be used for a variety of purposes [81]. RF generates a large number of decision trees, which the DT algorithm does not [82]. When RF predicts a new object based on specific attributes, each tree in the forest will give its own categorization result and ‘vote,’ and the forest’s total output will be the most taxonomic. In a regression problem, the RF output is the average of all decision tree outputs [83,84].

The number of trees in the classifier is controlled by the number of estimator parameters. The number of estimators and the maximum depth were rated at 500 and 5, respectively.

#### 2.6.2. K-Nearest Neighbors

The K-nearest neighbors (KNN) algorithm is a nonparametric classification algorithm, which means that no assumptions are made about the basic dataset. It is well known for its ease of use and effectiveness [85]. The data points are categorized into several classes in a labeled training dataset, allowing the class of the unlabeled data to be predicted.

Different variables determine which class the unlabeled data belongs to in classification. It is used to classify data in a given location based on the closest or nearby training samples. This method is employed because it is simple to implement and takes a short amount of time to compute. It calculates its nearest neighbors using a metric distance for continuous data [86]. The K-nearest neighbors are calculated for new input, and the majority of the neighboring data determines the new input’s classification.

The ‘K’ value is critical in identifying unlabeled data. When the value of k = 1 is used, the best outcomes are produced. The bounds, however, are overfitted when k = 1. The algorithm is too sensitive to noise when the value of ‘k’ is relatively tiny.

Larger ‘k’ values smooth the class boundaries, which may not be desirable because other classes’ points may be included in the neighborhood [87].

There are several methods for determining the values for ‘K’, but we used grid search to determine which number produced the best results, as well as the metric for calculating the distance between the locations. In the KNN algorithm, a certain value of K is fixed, which aids in the classification of the unknown tuple. The grid search algorithm returned k = 15 as well as the Euclidean distance between the points to be regarded as results: $d_{euclidean}=\sqrt{\sum_{i=1}^{n}{\left(x_{i}-y_{i}\right)}^{2}}$.

The computation cost is significantly higher because all computations are performed when the training data is classified rather than when it is met in the dataset. It is referred to as a “lazy” learning algorithm because it only stores training data and memorizes the dataset while being trained [88,89].

#### 2.6.3. Support Vector Machine

The support vector machine (SVM) is a binary classifier that assigns a class to new instances in a nonprobabilistic manner. It is a supervised kernel-based technique that first analyzes data with known classes before classifying unknown test samples. Intuitively, the hyperplane with the greatest distance to the nearest training data points of any class (so-called functional margin) achieves a decent separation because the larger the margin, the lower the generalization error of the classifier.

SVM can be implemented in a linear or nonlinear manner. Nonlinear SVM produces better results when the linear margin hyperplane does not yield a good match [90]. In order to improve the accuracy of the model, we used grid search to evaluate two hyperparameters in our model: *C* (regularization) is the penalty parameter, which reflects misclassification or error term, and *Gamma*, which specifies how far the calculation of the probable line of separation is influenced. The SVM optimization uses the misclassification or error term to determine how much error is acceptable. In this way, the trade-off between decision boundary and misclassification term can be managed. When C is high, it will accurately categorize all of the data points, but there is a risk of overfitting. When gamma is higher, nearby points have more effect; when gamma is low, far distant points are also taken into account when determining the decision border. As a result of the hyperparameter optimization, we used a linear SVM with C = 6 and gamma = 0.01.

#### 2.6.4. Artificial Neural Network

Artificial neural networks (ANNs) have been developed to simulate the human brain when it comes to processing information. The network is made up of many densely connected processing components (neurons) that operate in tandem to solve an issue. Neural networks learn by observing others. They cannot be programmed to do something specific. The samples must be carefully chosen to avoid time waste and potential malfunction of the network. Because they solve classification and prediction problems without making users aware of the processes within the algorithm, the learning process of ANNs is difficult to explain. ANNs can be implemented in supervised or unsupervised procedures. For the purposes of this study, we implemented supervised ANN. The network compares its outputs to the desired outputs after processing the inputs. Errors are subsequently propagated back through the system, causing the weights that regulate the network to be adjusted. As the weights are regularly changed, this process repeats again. During the training of a network, the same set of data is processed multiple times as the connection weights improve. Current commercial network development packages include tools for tracking the effectiveness of ANN in improving and predicting the correct answer. These capabilities allow the training process to continue, with the system only halting when it reaches a statistically desired level of accuracy. Some networks, on the other hand, never learn. This could happen because the input data lacks the exact information needed to generate the intended output. If there is not enough data for complete learning, networks will not converge. Ideally, there should be enough data to hold back a portion of it as a test. Data can be memorized by many multilayer networks with numerous nodes. To verify whether the network is simply memorizing its input in some insignificant way, supervised training must hold aside a batch of data that will be used to test the system once it has been trained. If a network fails to solve the problem, the designer must examine the inputs and outputs, the number of layers, the number of pieces per layer, the connections between layers, the summation, transfer, and training functions, and even the initial weights. An ANN first configures itself with the data’s general statistical tendencies. Later, it learns about other features of the data that may be suspect from a broad perspective. When the system has been properly trained and no more learning is required, the weights can be stopped if desired [91]. In this study, we applied a multilayer perceptron (MLP) as the ANN [34]. There are at least three levels of nodes in an MLP: an input layer, a hidden layer, and an output layer. Each node, except for the input nodes, is a neuron with a nonlinear activation function. Backpropagation is a supervised learning technique used by MLP during training. MLP is distinguished from a linear perceptron by its numerous layers and nonlinear activation. It can express the difference between data that is not linearly separable.

We chose the number of hidden layers in the network and configured the hyperparameters to increase the classifier’s accuracy as much as feasible.

Hyperparameters determine the network topology (for example, the number of hidden units) and how the network is trained (e.g., learning rate). Stochastic gradient descent (SGD) was chosen as the training optimization algorithm.

**Figure 3:**
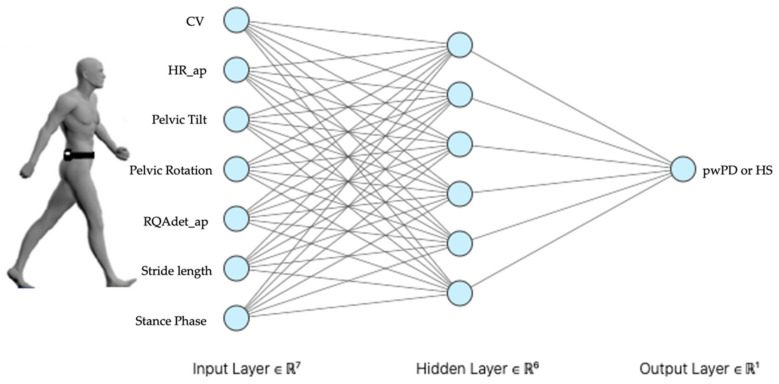
Multilayer perceptron (MLP). Our MLP with seven neurons in input layer, consequently features chosen by dimensionality reduction methods, six neurons in hidden layer, chosen by grid search method, classify our dataset in output layer to predict pathological or healthy subjects’ gait: CV, coefficient of variation of step length; HR ap, harmonic ratio anteroposterior; RQAdet_ap, % determinism in the recurrence quantification analysis anteroposterior; pwPD, people with Parkinson’s disease; HS, healthy subjects.

The hyperparameters were discovered using grid search. Figure 3 shows the network structure hyperparameters as a hidden layer with six neurons.

The uniform distribution is utilized for network weight initialization, and activation functions are employed to introduce nonlinearity to models.

The rectifier activation function (relu) is used in our scenario. When making binary predictions, the sigmoid function is employed in the output layer.

As regards the training algorithm hyperparameters, the learning rate, which determines how frequently a network updates its parameters, was set to 0.001, and the momentum was set to 0.8. Momentum helps to know the direction of the next step with the knowledge of the previous steps, and it helps to prevent oscillations.

The batch size was set to 80 (mini-batch size is the number of subsamples given to the network after which parameter update happens) with 100 epochs (number of epochs is the number of times the whole training data is shown to the network while training).

### 2.7. Evaluation of the Classification Performance

Each ML algorithm was run 10 times. Results have been reported in terms of mean ± standard deviations (SD). We calculated accuracy, precision, recall, and F1 score to assess the performance of the classifiers on the nonselected 22-features model and the selected 7-features model, as follows:

$$Accuracy=\frac{TP+TN}{FP+FN+TP+TN}$$

$$Precision=\frac{TP}{TP+FP}$$

$$Recall=\frac{TP}{TP+FN}$$

where *TP* represents the number of true positives predicted by the classifier, *TN* is the number of true negatives, *FP* is the number of false positives, and, consequently, FN is the number of false negatives.

$$F1\;score=\frac{2\times \left(Recall\times Precision\right)}{Recall+Precision}$$

We also calculated the area (AUC) under the receiver operating characteristics curve (ROC) and the generalization error as the difference between the errors in the training set accuracies and the error in the test set accuracy [92,93].

To assess the effectiveness of the selection procedure, we performed an independent sample *t*-test between the classification performance measures of the nonselected and selected model. Cohen’s d was also calculated to assess the effect sizes.

To assess the significance of the differences between the classification performance measures of each ML algorithm, a univariate ANOVA with Bonferroni’s post hoc comparison was performed using the type of ML algorithm as the factor [31,94]. Significance level was set at 95% for all the statistical tests.

The time performance of each ML algorithm was measured as the time of execution, including k-fold cross-validation and hyperparameter tuning, for both the 22-features unselected and 7-features selected models.

## 3. Results

### 3.1. Feature Selection

**Figure 4:**
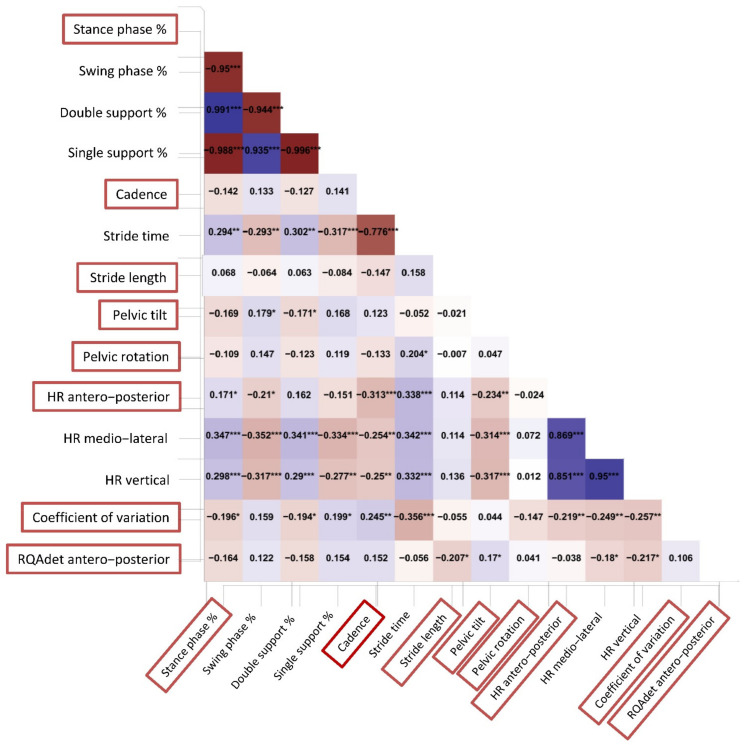
Partial correlation heatmap. Partial Pearson correlation analysis was performed to evaluate relationships among the initial set of features. A heatmap of the dataset’s characteristics showed different Pearson coefficients for every single variable. The deeper the red or blue color, the stronger the negative or positive correlation, respectively. In the decision process, we reduced the dimensionality of dataset according to the choice of which parameter we could keep that showed a partial correlation value ≥ 0.50 to prevent multicollinearity issues: *, *p* < 0.05; **, *p* < 0.01; ***, *p* < 0.001.

Out of the initial 22 gait parameters (Table 1), 14 showed significant differences in the independent sample *t*-test, regardless of gait speed (Table 1). We submitted to a clinical decision for those variables that reported a partial correlation value ≥ 0.50 with at least another feature (Figure 4). For example, stance phase was highly correlated with swing phase, double support, and single support. Because stance phase is complementary to swing phase and contains the information on support phases, we decided to maintain stance phase only in the final dataset. Another issue was the relationship between each gait stability index in the three spatial directions. We decided to maintain in the final dataset only HRap and RQAdetAP because of their clinical significance [5].

**Figure 5:**
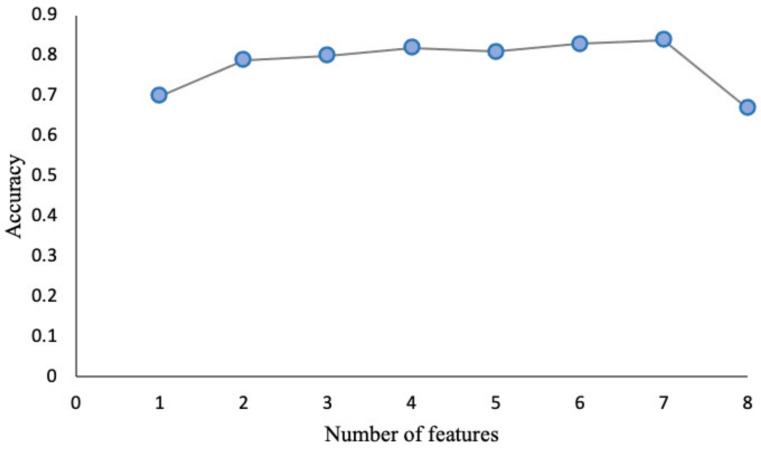
Sequential backward selection (SBS). To improve computational efficiency and reduce generalization error, the sequential backward selection algorithm aims to reduce the dimensionality of the initial feature subspace from N to K features with minimal model performance loss. To obtain the list of K features, sequentially remove features from a given features list of N features. By including seven characteristics of the dataset, excluding cadence, we maximized the performance of the algorithm, which, in our case, turned out to be KNN.

**Figure 6:**
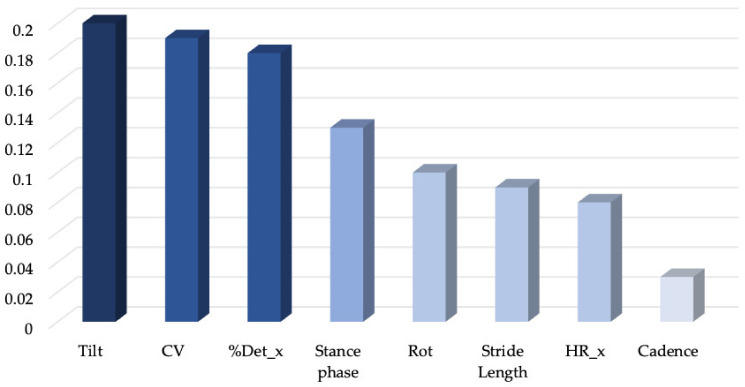
Random forest features importance. We analyzed features’ importance following implementation of a random forest of 1000 trees. As we showed in the histogram plot, cadence was evaluated by the random forest algorithm as the less important characteristic of our dataset, confirming the SBS result: HR_ap, harmonic ratio anteroposterior; RQAdet, % determinism in the recurrence quantification analysis anteroposterior; CV, coefficient of variation of the step length.

**Figure 7:**
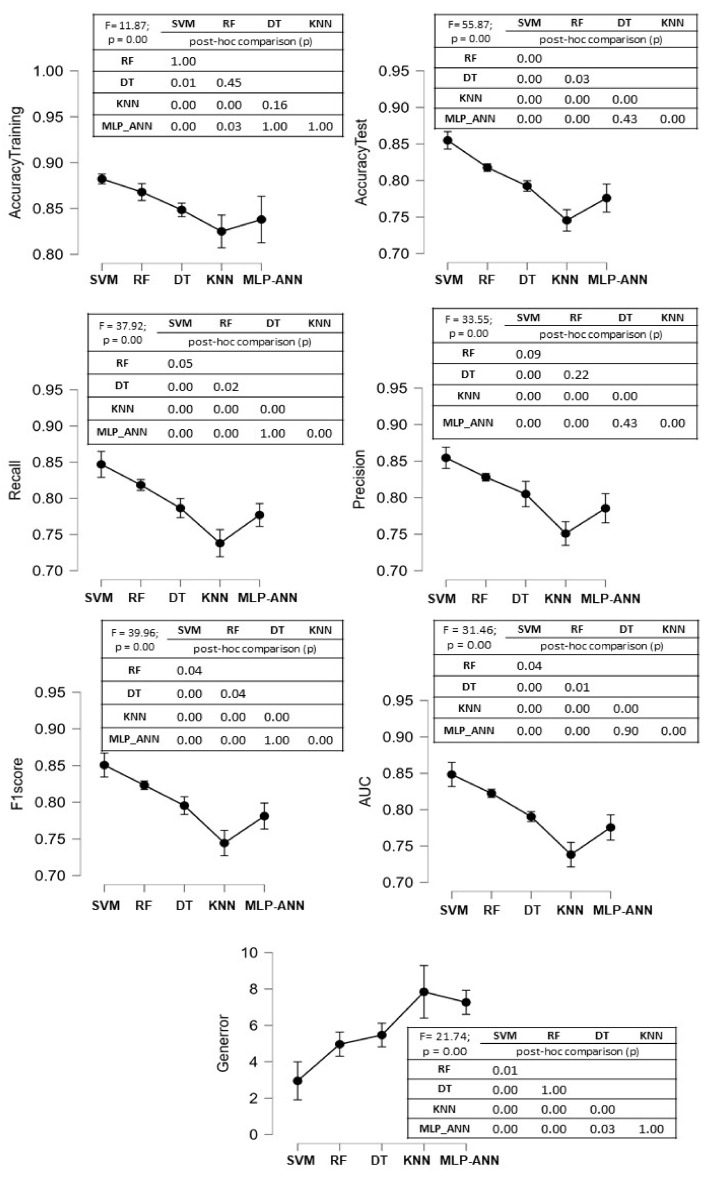
Comparison between the classification performance measures in the 7-features model. The mean and standard deviation values of the 10 runs for each performance measure for each ML algorithm are shown in the figure. The F values of the ANOVA procedure are reported, as well as the *p*-values of Bonferroni’s post hoc analysis.

Eight variables (Figure 5) were finally selected to input into SBS and random forest algorithms. Figure 6 and Figure 7 report the SBS and random forest selection algorithms results, respectively. SBS revealed that the performance capability of the classifier was excellent for seven out of the eight previously selected features, with the cadence lowering the accuracy of the dataset according to RF. As a consequence, a dataset of seven features was input into the supervised ML algorithms to assess their ability to classify the gait abnormalities of pwPD. A description of the selected gait parameters is described in the Supplementary Materials.

**Figure 8:**
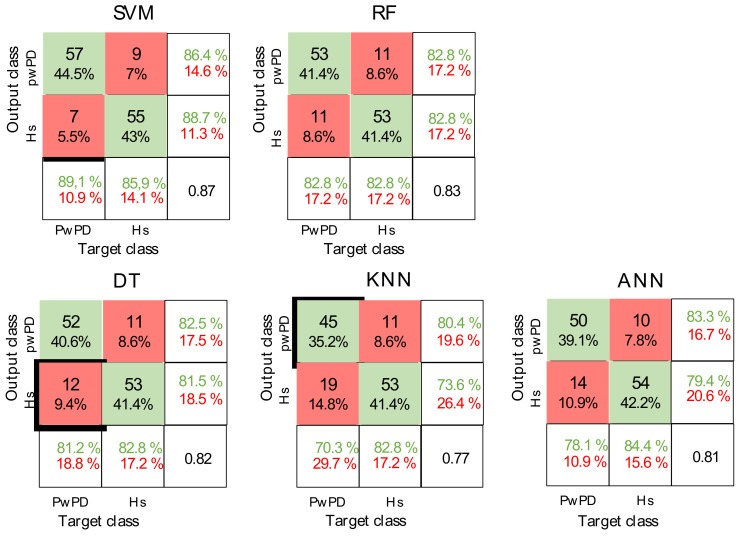
Confusion matrices. Representation of the confusion matrices evaluated for each algorithm after feature selection and during the run that displayed the highest accuracy value for each of them, The correct predictions are shown in green, while the wrong ones are shown in red.: PwPD, people with Parkinson’s disease; Hs, healthy subject; SVM, support vector machine; RF, random forest; DT, decision tree; KNN, K-nearest neighbor; ANN, artificial neural network.

A forest of 1000 trees was planted, and our eight features were ranked according to their relative value (Figure 8).

### 3.2. Supervised ML Algorithms Accuracy

Significant differences between the 22-features model and the three-levels 7-features selected model were found for all the performance measures of the ML algorithms. Notably, the generalization error was significantly lowered by the feature selection procedure (Table 2).

**Figure 9:**
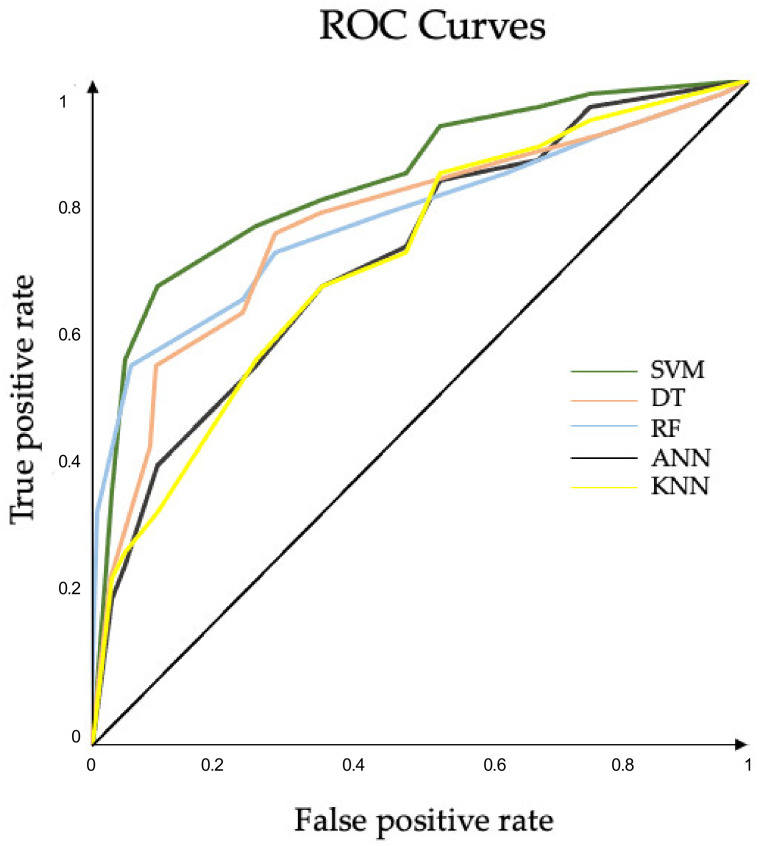
ROC curves. Plots of the runs that achieved the best AUCs in features selected approach for each ML algorithm. AUC SVM (0.877), AUC RF (0.827), AUC DT (0.820), AUC ANN (0.810), AUC KNN (0.778): ROC, receiver operating characteristic; AUC, area under curve; SVM, support vector machine; RF, random forest; DT, decision tree; KNN, K-nearest neighbor; ANN, artificial neural network. ROC curves for the maximum accuracies obtained by each classifier.

A significant main effect of the ML algorithms was found for all the performance measures. SVM outperformed DT, KNN, and ANN in all the classification performance measures and showed comparable precision values when compared with RF. In terms of precision and generalization error, no significant differences between RF and DT were found. Except for generalization error, no significant differences between DT and ANN were found in any of the classification performance measures (Figure 7). The confusion matrices and ROC curves for the maximum accuracies obtained by each classifier are reported in Figure 8 and Figure 9, respectively.

Following the feature selection procedure, the time performances of each ML algorithm decreased. KNN and DT returned results in less time. ANN took longer to return the results (Table 3).

## 4. Discussion

**Figure 10:**
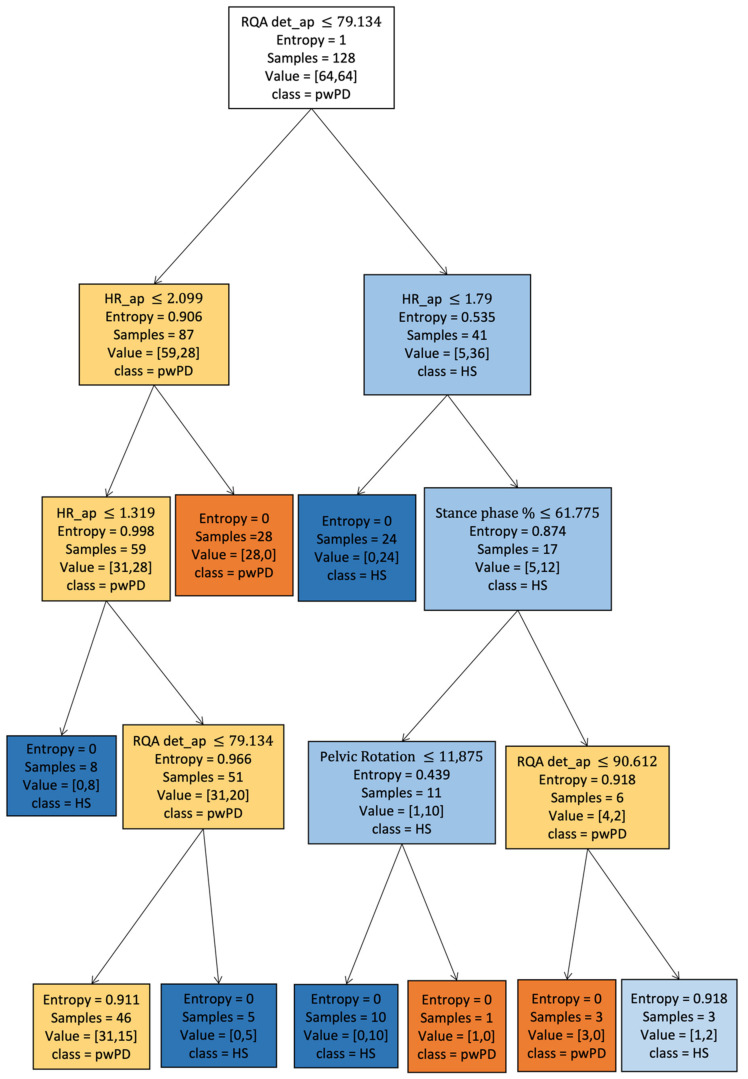
Decision tree graphical visualization. Graphical representation of our decision tree with maximum depth of five and used impurity criterion of entropy. In the decision-making process of the decision tree, we can observe the importance of the parameter RQAdet_ap to discriminate between Parkinson’s and healthy subjects in root node. As can be observed from the internal nodes, HR_ap, and RQAdet_ap are very important in the forecasting process: HR_ap, harmonic ratio anteroposterior; RQAdet_ap, % determinism in the recurrence quantification analysis anteroposterior; pwPD, people with Parkinson’s disease; HS, healthy subjects.

The main aim of this study was to determine which supervised ML algorithm can mostly accurately discriminate pwPD from a sample of speed-matched HS based on a selected set of gait features derived from a single lumbar-mounted IMU. The best ability to correctly classify pwPD and HSmatched has been reached by SVM, followed by RF and DT with similar classification performances (Table 2). These results further reinforce the applicability of SVM in gait prediction [20,22,95] while also highlighting the potential usefulness of tree-based methods such as RF and DT [96]. ML algorithms in gait analysis can be exploited with several aims. The advantages of using SVM for gait classification have been previously described [31,44] and rely on its performance ability in a tiny dataset [97] and its computational gain. However, because of the black-box nature of SVM, clinicians are unaware of the decisional processes besides the final classification, which makes it a suitable tool for automating diagnosis but limits the interpretation of the results. Furthermore, in this study, SVM showed higher computational cost in terms of time performance than tree-based algorithms. Because they are easy to explain and interpret, RF and DT could make clinicians participate in the decision process, provide information on the importance and relationships between the gait variables [98], and be exported in decision-making on patients (Figure 10). RF demonstrated good classification metrics using our data, despite being significantly different from SVM. Our results are consistent with those from other studies that described similar accuracy of the RF algorithm in distinguishing pwPD based on their disability level using IMUs-derived gait data [10], as well as in distinguishing the presence of freezing of gait [99,100]. Our study confirms the accuracy of RF as a useful algorithm to classify the gait abnormalities of pwPD from HS using trunk acceleration-derived data based on continuous walking tasks.

Although DT revealed similar classification metrics to ANN in this study, DT appears to fit better with trunk acceleration-derived gait data than ANN due to its computational gain (Table 3), lower generalization error (Table 2), and higher explainability. However, no information on the accuracy of the training set was provided in this study, and no feature selection was performed before the model was input into the ML models, exposing the results to an uncontrolled risk of bias. Because the performance of a classifier is dependent on the characteristics of the gait features, another source of difference between our results and those of Moon et al. could be represented by the use of preprocessed gait data from multiple IMUs placed at various localizations, which considered different gait features than did ours. Another issue about ANN could be linked to the methodology that we used in this study to find the best values for the hyperparameters. Because ANN encompasses a greater number of hyperparameters compared with the other ML algorithms that we investigated, a loss of accuracy of the model could have occurred. However, a deeper hyperparameter tuning for ANN could have led to a higher computational cost [101].

Although KNN achieved good performances in the training dataset, it revealed the worst classification performance and the higher generalization error in this study. The better performance of SVM and tree-based compared with the KNN algorithm could depend on the fact that, for low values of k, the algorithm is very sensitive to the presence of unseen outliers in the starting dataset. Our findings confirm the limitations of KNN, as reported by other studies on gait data based on technologies other than IMUs [102]. Other studies [103,104], conversely, reported good performance of KNN. Li et al. used KNN to classify pathological gait based on data from a Kinect camera, which provided gait features that are different from IMUs-derived signals. Demrozi et al. used KNN in predicting the freezing of gait in Parkinson’s disease patients, concluding that, due to its simplicity, KNN may be suitable for real-time classification using wearable devices. The differences in our results could be attributed to the different aims of the studies as well as the different applications of KNN. In the study by Demrozi et al., KNN was implemented on the training features solely, and a lower number of K was used. In this study, we presented results on both training and test data, emphasizing that KNN has a higher risk of overfitting than other algorithms. In addition, grid search was used to reduce the number of K. As a result, our KNN procedure had the lowest time performance when compared with the other ML algorithms, which is expected when using KNN, highlighting the lower generalizability of KNN to unknown data.

Other studies have examined the predictive power of ML algorithms on gait datasets for conditions such as Parkinson’s disease. For example, Caramia et al. [23] proposed a simultaneous analysis of the effects of IMU sensor localization on the extraction of path characteristics and compared the classification capacity of each configuration using a variety of machine learning techniques. Our work was evaluated using a different criterion, as our primary objective was to find a well-defined approach using our model for selecting features and optimizing hyperparameters to evaluate the classification performance of the algorithms we chose. Rehman et al. [1] and Moon et al. [36] investigated the classification performance of various ML algorithms in pwPD using gait data based on trunk accelerations. These studies, however, lacked a procedure for selecting the features to be input into the model. Conversely, we applied a multilevel feature selection approach combining statistical and domain expertise methods. When implementing ML models, reducing the number of features in the dataset to the sample size is a key point because of the risk of overfitting in the model prediction [105]. Combining different methods of feature selection with clinical perspectives can improve the accuracies of ML models. In this study, we subdued the model to clinical decisions, which acted as Occam’s razor, after the filter approach represented by the *t*-test and correlation analysis. Consequently, wrapped (SBS) and embedded (RF) methods were applied. As a result, we obtained a dataset of seven trunk acceleration-derived gait features to input into the ML models, including spatiotemporal parameters, pelvic kinematics, and three acceleration-derived gait indexes, namely HR, CV, and RQAdet, which have been recently described as accurate biomarkers of gait instability in pwPD [5]. Because gait speed is highly correlated with the other spatiotemporal and kinematic gait characteristics [51,68], we excluded its effects on the differences between pwPD and HS by matching procedures and by partial correlation analysis in the feature selection process to avoid the risk of multicollinearity. We further excluded gait cadence from the final dataset because of its detrimental effects on the SBS algorithm, as confirmed by RF. Abnormal cadence is usually described as an early characteristic of the gait of pwPD. Gait cadence intrinsically correlates with gait speed; hence, its exclusion from the dataset confirms the gait speed-independency of the final dataset. The remaining seven features that were input into the ML algorithms are representative of the range of gait abnormalities that pwPD suffer. The comparison with the unselected model, including the 22 preprocessed gait features, revealed the effectiveness of this feature selection approach in lowering the risk of overfitting in the ML classification metrics. Significant differences with large effect sizes were discovered between the models, and the model containing the seven selected features reduced the generalization error significantly, which was calculated as the difference between the error in the training and test set accuracies [92,93,106] (Table 2). To the best of our knowledge, this is the first time the effectiveness of a feature selection process providing a measure of generalizability in gait data studies has been reported. Furthermore, the low standard deviations of the mean values of the 10 runs we implemented for each ML algorithm confirmed the effectiveness of the k-fold cross-validation method in terms of reliability (Table 2). However, in this study, stratification of the sample based on the Hoehn and Yahr scores was not possible because of the small sample size. Consequently, the gait feature importance provided by RF is probably biased by the presence of subjects at the initial stages of the disease who typically do not show axial impairments or evident postural instability. Furthermore, the level of disability of people with Parkinson’s disease (pwPD) can influence the number of discriminating features generated by RF based on IMU localization [10]. In this way, it is not surprising that CV and RQAdet reached higher importance than HR or pelvic rotation. RQAdet and CV have been described as altered in pwPD regardless of the Hoehn and Yahr score, whereas other parameters, such as HR and pelvic rotation, have been described as excellent biomarkers of gait instability in subjects with higher levels of disability, reflecting the appearance of axial impairment. Therefore, it is auspicial that the methods used in this study could be implemented in studies including larger sample sizes to explore the ability of ML to classify the disease stages of pwPD. Another issue with our study is the unequal gender distribution in our sample. In this way, a further feature selection could have been used to include those that distinguish pwPD and HS regardless of gender. However, gender is not a primary predictor of gait impairment in Parkinson’s disease [107,108,109], impacting gait speed only [110,111]. Because we excluded the effect of gait speed on the differences between subjects with Parkinson’s disease and healthy subjects in this study, the contribution of this additional feature selection to our study would have been moderate. A study limitation was that the features were not extracted to reduce the dataset’s dimensionality before analyzing the effectiveness of the various algorithms [112]. However, we aimed to emphasize clinical explainability by selecting the input gait features through a combination of statistical and experience-based methods. From a clinical perspective, identifying the single gait features could provide better information to tailor treatments to patients’ characteristics, compared with the comprehensive subsets of features that result from feature extraction methods, such as principal component analysis. Another issue of this study was represented by using grid search as a method [113] for optimizing the hyperparameters of all the analyzed ML algorithms. Although grid search is an effective method for optimizing the hyperparameters, other methods of tuning could have resulted in higher accuracies of some of the ML algorithms. To test this hypothesis, the methodological model of conducting ML algorithms that we proposed should be tested by applying a range of tuning methods for each ML algorithm. Furthermore, other ML algorithms could have been implemented in our study, such as deep learning. However, they often need a large amount of data to work efficiently and are not suitable for relatively small datasets such as [114]. As a consequence, we focused on the five most-used algorithms in gait analysis studies using wearable devices on subjects with Parkinson’s disease that have been shown to work efficiently on small datasets [31]. We, therefore, conclude that in addition to the optimal ability to classify SVM, the positive results for the tree-based algorithms support previous research [96] and could place more focus on them by opening a new chapter in ML-supervised categorization for gait analysis in patients with pathology. Their simplicity of understanding and explanation could make them a useful tool in diagnosing neurodegenerative diseases, and future research could focus on finding the best tree-based model for gait analysis datasets.

## 5. Conclusions

SVM outperformed the other ML algorithms in terms of classification metrics (test accuracy = 0.86; F1 score = 0.85; AUC = 0.85) and generalizability (generalization error = 2.95%) in classifying the gait impairment of pwPD compared with speed-matched healthy subjects, using a selected dataset of gait features based on lower trunk acceleration data. Although significantly lower than SVM, tree-based algorithms revealed good classification performances with low generalization errors (RF: test accuracy = 0.86; F1 score = 0.85; AUC = 0.85), and lower computational demand than SVM. ANN was similar to DT in terms of classification metrics but showed significantly higher generalization error (7.26%) than tree-based algorithms and SVM and higher computational demand than the other ML algorithms. Despite the fact that KNN showed the fastest time performance, its classification metrics were the lowest.

In this study, we proposed a feature selection procedure based on the combination of filter, wrapper, embedded, and domain-specific methods that was effective in lowering the risk of overrepresenting multicollinear gait features in the model, resulting in a lower risk of overfitting in the test performances by increasing the explainability of the results at the same time. Because of their accurate results, their simplicity of understanding, and explanability, DT and RF algorithms could represent useful tools for the comprehension of gait disorders by making clinicians participate in the decision process. This is the first time that the accuracy and generalizability of the most commonly used ML algorithms in classifying pwPD gait abnormalities based on gait data from a single lumbar-mounted IMU have been compared. The findings of this study could be used to incorporate machine learning algorithms into software that processes gait data from lumbar-mounted IMUs. Future research could focus on finding the best tree-based model for classification and prediction problems in gait analysis.

## Figures and Tables

**Table 1:** Features selection: independent sample *t*-test. We performed an independent sample *t*-test to analyze which features showed significant differences, regardless of gait speed: pwPD, persons with Parkinson’s disease; HR, harmonic ratio; RQArec, %recurrency in the recurrence quantification analysis; RQAdet, % determinism in the recurrence quantification analysis; CV, coefficient of variation of the step length.

	pwPD		Hsmatched		*p*	Cohen’s d
	Mean	SD	Mean	SD		
Gait speed (m/s)	0.76	0.21	0.81	0.22	0.171	0.233
Stance phase (%)	60.84	2.13	62.19	3.17	0.004	0.498
Swing phase (%)	39.12	2.14	37.93	3.21	0.011	0.438
Double support (%)	10.86	2.13	12.09	3.17	0.008	0.453
Single support (%)	39.09	2.13	37.84	3.19	0.008	0.457
Cadence (steps/min)	104.06	18.10	88.31	13.23	<0.001	0.994
Stride time (s)	1.22	0.18	1.37	0.18	<0.001	0.839
Stride length (m)	0.91	0.19	1.05	0.17	<0.001	0.783
% stride length (% height)	57.35	22.08	62.79	11.67	0.148	0.288
Pelvic tilt (°)	3.46	1.45	2.74	0.71	<0.001	0.624
Pelvic obliquity (°)	4.04	1.88	4.01	1.53	0.923	0.017
Pelvic rotation (°)	5.26	2.98	6.53	3.57	0.025	0.383
HRap	1.64	0.30	2.07	0.54	<0.001	0.973
HRml	1.60	0.27	1.96	0.42	<0.001	0.996
HRv	1.64	0.30	2.09	0.54	<0.001	1.008
RQArec_ap	21.09	17.85	4.93	3.80	<0.001	1.257
RQArec_ml	15.53	14.03	4.35	5.01	<0.001	1.064
RQArec_v	37.17	193.60	3.13	3.22	0.144	0.250
RQAdet_ap	70.29	31.20	38.40	27.46	<0.001	1.086
RQAdet_ml	67.45	33.37	36.61	26.26	<0.001	1.028
RQAdet_v	69.02	29.39	23.54	18.89	<0.001	1.843
CV	39.04	19.65	26.90	10.91	<0.001	0.765

**Table 2:** Comparison of the classification performances of the supervised machine learning accuracies between the 22-features and the 7-features models.

		SVM		RF		DT		KNN		MLP-ANN	
		22 Features	7 Features	22 Features	7 Features	22 Features	7 Features	22 Features	7 Features	22 Features	7 Features
**Accuracy Training**	Mean*_10runs_* (SD)	0.91 (0.01)	0.88 (0.01)	0.89 (0.01)	0.87 (0.01)	0.89 (0.02)	0.85 (0.01)	0.86 (0.03)	0.82 (0.02)	0.87 (0.02)	0.84 (0.03)
	*p* (d)	0.00 (2.48)		0.00 (1.59)		0.00 (2.13)		0.00 (1.59)		0.03 (1.03)	
**Accuracy Test**	Mean*_10runs_* (SD)	0.81 (0.01)	0.86 (0.02)	0.79 (0.01)	0.82 (0.01)	0.76 (0.02)	0.79 (0.01)	0.68 (0.02)	0.74 (0.02)	0.74 (0.02)	0.77 (0.03)
	*p* (d)	0.00 (3.17)		0.00 (2.68)		0.00 (1.79)		0.00 (2.99)		0.01 (1.32)	
**Precision**	Mean*_10runs_* (SD)	0.80 (0.01)	0.85 (0.02)	0.79	0.83	0.76 (0.03)	0.81 (0.02)	0.68 (0.02)	0.75 (0.02)	0.74 (0.03)	0.78 (0.03)
	*p* (d)	0.00 (3.12)		0.00 (2.94)		0.00 (1.75)		0.00 (3.03)		0.00 (1.19)	
**Recall**	Mean*_10runs_* (SD)	0.80 (0.02)	0.85 (0.03)	0.78 (0.03)	0.82 (0.01)	0.75 (0.03)	0.79 (0.02)	0.69 (0.02)	0.74 (0.03)	0.74 (0.02)	0.77 (0.03)
	*p* (d)	0.00 (2.42)		0.01 (1.45)		0.00 (1.67)		0.00 (2.08)		0.00(1.59)	
**F1score**	Mean*_10runs_* (SD)	0.80 (0.01)	0.85 (0.02)	0.78 (0.03)	0.82 (0.01)	0.75 (0.03)	0.80 (0.02)	0.68 (0.02)	0.74 (0.02)	0.74 (0.03)	0.78 (0.02)
	*p* (d)	0.00 (2.65)		0.00 (2.01)		0.00 (1.88)		0.00 (2.58)		0.02 (1.54)	
**AUC**	Mean*_10runs_* (SD)	0.80 (0.01)	0.85 (0.02)	0.78 (0.03)	0.82 (0.01)	0.75 (0.03)	0.79 (0.01)	0.68 (0.02)	0.74 (0.02)	0.73 (0.03)	0.77 (0.02)
	*p* (d)	0.00 (2.75)		0.00 (1.86)		0.00 (1.85)		0.00 (2.43)		0.00 (1.56)	
**Generalization Error (%)**	Mean*_10runs_* (SD)	9.35 (1.31)	2.95 (1.46)	9.93 (1.16)	4.96 (0.92)	12.44 (1.31)	5.47 (0.91)	17.70 (0.69)	7.84 (2.02)	12.80 (2.51)	7.26 (0.92)
	*p* (d)	0.00 (4.62)		0.00 (4.71)		0.00 (6.17)		0.00 (6.53)		0.00 (2.93)	

SVM, support vector machine; RF, random forest; DT, decision tree; KNN, k-nearest neighbor; MLP-ANN, multilayer perceptron artificial neural networks; SD, standard deviation; d, Cohen’s effect size.

**Table 3:** Time performances of each ML algorithm. SVM, Support vector machine; RF, Random Forest; DT, Decision tree; KNN, K-Nearest-Neighbor; MLP-ANN, Multilayer Perceptron—Artificial neural network.

	SVM	KNN	MLP-ANN	DT	RF
22 features (unselected model)	08:12:12	00:12:53	16:34:00	00:45:16	01:45:01
7 features (Selected model)	05:25:29	00:08:09	10:37:20	00:15:58	01:10:29

## Data Availability

The data presented in this study are available on request from the corresponding author and stored in a password-protected PC located in the Department of Medico-Surgical Sciences and Biotechnologies, University of Rome Sapienza.

## Supplementary Materials

The following supporting information can be downloaded at: https://www.mdpi.com/article/10.3390/s22103700/s1, Sequential Backward Selection programming code, Description of the selected gait variables in the machine learning model, List of abbreviations in the manuscript.
