# Supplementary material for: Machine Learning Approach to Support the Detection of Parkinson’s Disease in IMU-Based Gait Analysis
Source: Sensors (Basel). 2022 May 12;22(10):3700. doi: 10.3390/s22103700 (PMC9148133; doi:10.3390/s22103700)
Supplement: Supplementary file 1 [file sensors-22-03700-s001.zip › sensors-1691681-supplementary.pdf]

SUPPLEMENTARY MATERIAL

Sequential Backward Selection programming code.

```
from sklearn.base import clone
from itertools import combinations
import numpy as np
from sklearn.cross_validation import train_test_split
from sklearn.metrics import accuracy_score
class SBS ()
def_init_ (self, estimator, k_features,
scoring=accuracy_score
test_size=0.20, random_state =1):
self.scoring = scoring
self.estimator = clone(estimator)
self.k_features = k_features
self.test_size = test_size
self.random_state = random_state

def fit (self, X, y):
X_train, X_test, y_train, y_test = \
train_test_split(X, y, test_size=self.test_size,
random_state=self.random_state)

dim= X_train.shape[1]
self.indices = tuple (range(dim))
self.subsets_ = [self.indices_]
score = self. _calc_score(X_train, y_train,
X_test, y_test, self.indices_)

self.scores_ = [score]

while dim > self.k_features:
scores = []
subsets = []

for p in combinations (self.indices_, r=dim-1):
score = self. _calc_score(X_train, y_train,
X_test, y_test, p)

scores.append(score)
subset.append(p)

best= np.argmax(scores)
self.indices_ = subsets[best]
self.subsets_.append(self.indices_)
dim-=1

self.scores_.append(scores[best])
self.k_score_ = self.scores_[-1]

return self
```

```
def transform (self, X):
return X [:  , self.indices_]
def_calc_score(self, X_train, y_train,
X_test, y_test, indices):
self.estimator.fit(X_train[: , indices], y_train)
y_pred = self.estimator.predict (X_test[:, indices])
score = self.scoring(y_test, y_pred)
return score
```

Description of the selected gait variables in the machine learning model.

Feature name	Feature description
Stance Phase (%)	The stance phase accounts for nearly 60% of the gait cycle. The time while the foot is in contact with the ground and the leg is bearing weight is referred to as "contact time." This phase begins when the ipsilateral foot makes initial contact with the ground and ends when the ipsilateral foot exits the ground. In healthy subjects, during an average walking speed, the stance phase lasts roughly 0.6 seconds.
Stride length (m)	During walking, is the distance between ipsilateral heel contact and the next ipsilateral heel contact (i.e., right-to-right, or left-to-left heel contact). Stride length can be calculated based on cadence and walking speed.
Pelvic Tilt (°)	Pelvic tilt is a position-dependent measure defined as the angle formed by a line drawn from the middle of the sacral endplate to the centre of the bifemoral heads, as well as the vertical axis. (Positive angular values imply a pelvic anterior tilt, while negative angular values suggest a pelvic posterior tilt)
Pelvic Rotation (°)	The horizontal rotation angle of the pelvis in the global horizontal plane, in which the pelvis is identified between the right and left anterior superior iliac spine markers, was described as pelvic rotation (negative angular values indicate pelvis internally rotated, positive values indicate a pelvis externally rotated)
Harmonic Ratio antero-posterior	The Harmonic Ratio is calculated through a spectral analysis of lower trunk accelerations-based metric that is often used to assess gait quality. The Harmonic Ratio is a metric for determining the smoothness of trunk acceleration during walking. In this study, the Harmonic Ratio in the anteroposterior direction (HR_{AP}) was calculated as the ratio between the sum of the amplitudes of the first 10 even harmonics and the sum of amplitudes of the first 10 odd harmonics as follows: $HR_{AP} = \frac{\sum_i A_{i*2}}{\sum_i A_{i*2-1}}$

	where A_i represents the amplitudes of the first 20 even harmonics and A2i–1 represents the amplitudes of the first 20 odd harmonics. A discrete Fourier transform was used to break down the trunk accelerations of each stride into individual sinusoidal waveforms.<br>The HR measures the harmonic composition of these accelerations for a given stride, with a higher HR indicating more smooth walking.
Coefficient of variation	The standard deviation divided by the mean is the coefficient of variation. The Coefficient of Variation (CV) for stride length was calculated as CV=100SD/mean, where SD is the standard deviation over all step lengths for each subject and mean is the mean step length. The greater the CV, the greater the step length variability.
RQAdet antero-posterior	The recurrent quantification analysis (RQA) is the foundation of percentage determinism.<br>RQA is a non-linear technique that can reveal useful information regarding system dynamics patterns and structures. Based on the building of recurrence graphs, it gives a characterisation of a range of features of a given time series, including the quantification of deterministic structures and non-stationarity. The structure of the recurrence matrix is quantified using RQA variables. Percent Determinism (%det) can be calculated to determine how frequently a trajectory revisits similar positions in state space (time dependent) and is measured as the percentage of recurring points in diagonal line structures parallel to the main diagonal. Based on the reconstruction of recurrence plots, RQA_{detAP} was calculated through the nearest-neighbor method by embedding acceleration and angular velocity data in a range of embedding dimension (m) 2-10 considering an m = 5 as the optimal one. False neighbors' analysis was performed using values of Rtol = 17 and Atol = 2. The second recurrence parameter τ was calculated from the first minimum of the average mutual information function (AMI). We considered a range of τ = 7-18 in this study, and we computed 10 samples as the time delay for the first minimum of the AMI function.<br>$$\% \det = \frac{\sum_{l=lmin}^{N} lP(l)}{\sum_{l=1}^{N} lP(l)}$$<br>Where *P(l)* is the frequency distribution of the lengths *l* of the diagonal lines in the recurrence matrix (i.e., it counts how many patterns have length *l*)

LIST OF ABBREVIATIONS IN THE MANUSCRIPT.

ANN, artificial neural network;
ap, antero-posterior direction of the acceleration pattern;
AUC, area under curve;
CV, coefficient of variation of step length;
DT, decision tree;
FN, false negative rate;
FP, false positive rate;
H&Y, Hoehn and Yahr staging scale;
HR, harmonic ratio;
HS, healthy subjects;
IMU, inertial measurement unit;
KNN, K-nearest-neighbours;
ML, machine learning;
ml, medio-lateral direction of the acceleration pattern
MLP, multilayer perceptron;
PD, Parkinson disease;
pwPD, people with Parkinson's disease;
ReLU, rectified linear unit;
RF, random forest;
ROC, receiver operating characteristic;
RQAdet, percentage of determinism in the recurrence quantification analysis;
RQArec, percentage of recurrence in the recurrence quantification analysis;
SBS, sequential backward analysis;
SGD, stochastic gradient descend;
SVM, support vector machine;
TN, true negative rate;
TP, true positive rate;
v, vertical direction of the acceleration pattern.
